# Enhancing family physicians’ clinical research skills at Cairo University: an evaluation of knowledge transfer and perception of an online training

**DOI:** 10.1186/s12909-025-07090-1

**Published:** 2025-04-22

**Authors:** Saeed Soliman, Marwa M. Ahmed, Nadia M. Tawfik

**Affiliations:** Department of Family Medicine, Faculty of Medicine, Cairo University, Cairo, Egypt

**Keywords:** Clinical, Research, Family medicine, Education

## Abstract

**Background:**

Clinical research is an important academic function for maximizing the health benefits of interventions. This growing field requires creative trials to build expertise for health researchers and practitioners simultaneously.

**Objective:**

The purpose of this research was to assess the impact of clinical research training on the knowledge of family medicine postgraduate trainees.

**Methods:**

A quasi experimental study was conducted on 49 family medicine master’s and MD candidates in the family medicine department, Cairo university. Knowledge about clinical research was assessed before and after enrollment in the “Fundamentals of Clinical Research” online course. The trainees’ feedback about the course was assessed using an evaluation form.

**Results:**

49 participants’ responses were analyzed. They had a mean (± SD) age of 35.4 (± 8.7) years and 10.2 (± 8.7) years of practice. The majority were female (93.9%). Over 60% were engaged in clinical research projects. Feedback showed high satisfaction and pre-post comparison showed statistically significant increased knowledge scores in most areas, except research questions.

**Conclusions:**

The “Fundamentals of Clinical Research” training course increased knowledge scores among participants, and high satisfaction was reported. However, further exploration is needed to address the long-term impact on their career benefits and clinical decision skills.

## Introduction

Clinical research is increasingly being recognized as an important academic function for maximizing the interventions’ health benefits [1]. 

A research training course is an essential item of medical education and a crucial opportunity to develop future physicians and healthcare providers with excellent research skills [2]. 

Previous studies reported that research experience throughout the residency period of different specialties is strongly associated with future research achievements [3]. The research training courses project entail an understanding of various phases of the research project, starting from formulating a research question through conducting an efficient literature review and appraisal, developing the appropriate study methodology, as well as accomplishing data collection and analysis within a specified time frame, and finally acquiring and practicing the skills of manuscript writing [4]. Additionally, research training courses help health care professionals to develop critical thinking and to evaluate literature efficiently [5, 6]. Preparing physicians from different healthcare specialties by inculcating adequate information and research skills fosters a positive attitude towards research among physicians at all stages of their careers [6]. 

Consequently, there is a need for effective research courses and programs at all postgraduate levels, which can be vital in making a workforce of experienced researchers in family medicine practice. Accordingly, we have developed a “Fundamentals of Clinical Research” short course that targets both family medicine master’s and MD candidates.

Despite the presence of some postgraduate research educational programs, limited published reports illustrate, in detail, the development of the programs, and there is less research evidence of their educational impact [7–9]. Therefore, this study aimed to explain the course development processes and report on training evaluations and to assess the impact of the “Fundamentals of Clinical Research” course on the knowledge of family physicians.

## Methods

### Study design

Quasi experimental (pretest - posttest) study design.

### Settings, sampling, and participants

This study was performed on family medicine master’s and MD (medical doctorate) candidates during the academic year 2023–2024 in the family medicine department, Cairo University, Egypt.

The eligibility criteria were all the candidates enrolled in “Fundamentals of Clinical Research” training and who agreed to participate in the study.

Sample size and sampling procedures:

Sample size has been calculated using Epi Info *7*, based on assumptions from similar research [10] of improved knowledge from 22.8 to 85.4% at 80% power and 0.05 significance level. The minimum required sample size was 24. However, our sample size was further increased as a convenient sampling technique was adopted. All participants of the training were invited to participate in the evaluation, ending in 49 and 40 participants at pretest and posttest, respectively.

### Development of “fundamentals of clinical research” course

The development of “Fundamentals of Clinical Research” online course followed a logical and systematic method to ensure its relevance, effectiveness, and comprehensiveness. The process was categorized into four key phases:

#### Needs assessment phase

This phase involved identifying the knowledge gaps among postgraduate trainees regarding clinical research methodologies. A survey was conducted among residents and early-career physicians to assess their familiarity with clinical research concepts, common challenges they faced, and their perceived need for structured training in research skills. The results highlighted a lack of confidence in study design, statistical analysis, and scientific writing, justifying the need for a structured course.

#### The core team development that determined the scope of this short course

A multidisciplinary team was formed, consisting of experienced faculty members from the Family Medicine Department with expertise in clinical research, medical statistics, and medical education. This team was responsible for defining the course scope, setting learning objectives, and structuring the content to align with the needs assessment findings.

#### Course content development

The course was designed as a concise yet comprehensive program covering essential aspects of clinical research. The core team developed a structured curriculum divided into three modules, each focusing on a key component of research training. The content included practical examples, case studies, and interactive exercises to facilitate application-based learning.

#### Review of module content

To ensure the accuracy and relevance of the course material, each module underwent a rigorous review process by faculty members with expertise in clinical research. Feedback was gathered and used to refine the content before finalizing the course structure.

The course was delivered online during the academic year 2023–2024 as pre-recorded lectures supported with many helpful materials about learning clinical research within one month through 3 modules. The lectures were presented by different family medicine staff with experience in clinical research.

Topics of the online sessions:

Module 1: Research questions and research methods.

- Introduction to Clinical Research.[11]
- Ethical Considerations in Clinical Research [12].
- Study Design in Clinical Research [13].

Module 2: Basics of medical statistics.

- Biostatistics in Clinical Research [14].
- Data Collection and Management [15].

Module 3: Scientific writing.

- Protocol Development and Writing [16].
- Manuscript writing [16].
- Good Clinical Practice Guidelines [17].

### Development of assessment tools

To evaluate the effectiveness of the course and assess knowledge acquisition among participants, a structured assessment strategy was developed. This included:

1. Pretest and Posttest Assessments:

- A pretest was administered before the start of the course to assess baseline knowledge of clinical research concepts.
- A posttest was conducted after the completion of the course to measure knowledge gains and assess the effectiveness of the instructional materials.
- The questions in both assessments were designed to evaluate understanding of study design, ethical considerations, statistical analysis, and scientific writing.

2. Evaluation Form for Trainee Feedback:

- A post-course evaluation form was administered to collect feedback from participants regarding course content, delivery format, and overall satisfaction.
- The evaluation focused on the clarity of instruction, relevance of the materials, engagement of the learning process, and the applicability of acquired knowledge in clinical practice.

3. Questionnaire Development and Validation:

- The assessment questionnaires were developed after reviewing relevant literature on research training programs [18–20] to ensure validity and relevance.
- The questionnaires were reviewed by two faculty members from the Family Medicine Department with expertise in clinical research and medical education. Based on their feedback, modifications were made to improve clarity and comprehensiveness.
- A pilot study were conducted with seven candidates to test the applicability and reliability of the questionnaires. The data collected from this pilot study was used for refinement but was not included in the final analysis.

The final assessment tool consisted of three sections:

1. **Demographic Data**: Including age, sex, years of practice, and academic qualifications.
2. **Knowledge Assessment**: Focused on components of clinical research, study designs, research methodology, and current involvement in research activities.
3. **Course Evaluation**: Available only in the posttest to collect feedback on the training experience.

The systematic design of both the curriculum and assessment methods ensured that the course effectively addressed the learning needs of postgraduate trainees while also allowing for measurable evaluation of its impact on their research competencies.

### Study procedures

The training course was posted through the family medicine department official emails to invite all master’s and MD candidates to register as a mandatory training part to complete the requirements for getting a family medicine postgraduate degree.

When candidates enrolled in the course, a pretest were required to be filled after obtaining an informed consent to enroll in the study.

After one month from enrollment (the training duration), a post test and an evaluation form was required to be filled in by the candidates.

### Statistical analysis of data

The data collected were cleaned in Excel 365. Simple descriptive statistics (arithmetic mean and standard deviation) were used for a summary of quantitative data and frequencies were used for qualitative data, and Mann-Whitney U test was used to test difference in paired score medians (pretest and posttest). All analyses were performed using Stat ^®^ software, version 18.

All P-values less than 0.05 were considered significant.

### Ethical considerations

All participants provided informed written consent to participate. The study protocol was approved by research ethics committee (REC) number (N-404-2023).

## Results

A total of 49 participants completed the pre-training survey and pre-test; of them 40 completed the post-test. Table 1 provides information on the baseline characteristics of participants in the research training course, the mean (± SD) age of the participants was 35.4 (± 8.7) years. On average, the participants had 10.2 (± 8.7) years of practice. The majority of participants were female, accounting for 93.9%, while only 6.1% were male. In terms of scientific degrees, the participants were divided between MD students/trainees (51.0%) and master’s degree students/trainees (49.0%). 61.2% of participants reported being currently engaged in clinical research projects while undertaking this training course, whereas 38.8% were not. When asked about their attendance of previous courses or training in Clinical Research, 53.1% of participants answered positively, while 46.9% responded negatively.

**Table 1:** Baseline characteristics of research training course participants (*n* = 49)

	Mean (± SD) or Number (%)
**Age**	35.4 (8.7)
**Years of practice**	10.2 (8.9)
**Gender**	
Female	46 (93.9%)
Male	3 (6.1%)
**Scientific Degree**	
MD student /trainee	25 (51.0%)
Master’s degree student / trainee	24 (49.0%)
**Are you doing clinical research in the current period?**	
No	19 (38.8%)
Yes	30 (61.2%)
**Did you attend previous courses/ training in Clinical Research?**	
No	23 (46.9%)
Yes	26 (53.1%)

Regarding post-training surveys and feedback, Table 2 shows that 45% of students spent 2–5 hours and another 45% spent more than 5 hours to finish the online research course, while 10% spent less than 2 hours. Most students agreed or strongly agreed that the course content was clear and easy to understand (77.5%) and provided applicable theoretical information (79.0%). In terms of practical examples, 75% of students agreed or strongly agreed that the course provided them. Furthermore, 67.5% of students agreed or strongly agreed that the course prepared them to do clinical research. A significant portion of the students (87.5%) agreed or strongly agreed that they learned new things in the course, and 80% found the course to be relevant to their work in clinical research. The majority of students (77.5%) also agreed or strongly agreed that the course material challenged them to think about the topic in a different way. Additionally, 92.5% of students agreed or strongly agreed that they would be able to use the knowledge and skills gained in their future work, and the online format of the course was found to facilitate learning by 90%. Most students (85%) agreed or strongly agreed that it was easy to navigate through the course content, and 75% felt that the course met their expectations. The most useful module was identified as “Scientific Writing” by 60% of respondents. All students recommended the course to others. Overall, the course was rated as useful or very useful by 90% of the students, and their satisfaction levels were high, with 90% expressing ‘satisfied” or “very satisfied”.

**Table 2:** Summary of post-training survey responses (*n* = 40)

	Frequency (%)
**Hours did you spend on the course**:	
2–5 h	18 (45.0%)
>5 h	18 (45.0%)
Less than 2 h	4 (10.0%)
**The content of the course was clear and easy to understand**	
Agree	15 (37.5%)
Disagree	1 (2.5%)
Neutral	8 (20.0%)
Strongly Agree	16 (40.0%)
**The course provided applicable theoretical information**	
Agree	15 (37.5%)
Neutral	8 (20.0%)
Strongly Agree	17 (42.5%)
**The course provided practical examples**	
Agree	20 (50.0%)
Disagree	2 (5.0%)
Neutral	8 (20.0%)
Strongly Agree	10 (25.0%)
**The course has prepared me to do clinical research**	
Agree	16 (40.0%)
Disagree	3 (7.5%)
Neutral	9 (22.5%)
Strongly Agree	11 (27.5%)
Strongly Disagree	1 (2.5%)
**I learned new things in the course**	
Agree	15 (37.5%)
Neutral	5 (12.5%)
Strongly Agree	20 (50.0%)
**The course was relevant to my work in clinical research**	
Agree	17 (42.5%)
Disagree	1 (2.5%)
Neutral	7 (17.5%)
Strongly Agree	15 (37.5%)
**The course material challenged me to think about the topic in a different way**	
Agree	21 (52.5%)
Disagree	2 (5.0%)
Neutral	7 (17.5%)
Strongly Agree	10 (25.0%)
**I shall be able to use the knowledge and skills I have gained for improving my future work in research**	
Agree	21 (52.5%)
Neutral	3 (7.5%)
Strongly Agree	16 (40.0%)
**The fact that the course was fully online facilitated my learning experience**	
Agree	21 (52.5%)
Disagree	1 (2.5%)
Neutral	3 (7.5%)
Strongly Agree	15 (37.5%)
**It was easy to navigate through the course content**	
Agree	16 (40.0%)
Disagree	1 (2.5%)
Neutral	5 (12.5%)
Strongly Agree	18 (45.0%)
**The course met my expectations**	
Agree	16 (40.0%)
Disagree	1 (2.5%)
Neutral	9 (22.5%)
Strongly Agree	14 (35.0%)
**What was the most useful module in the course**	
Introduction to Biostatistics	6 (15.0%)
Introduction to research	10 (25.0%)
Scientific writing	24 (60.0%)
**Do you recommend the course to others?**	
Yes	40 (100.0%)
**Overall**,** how would you rate the usefulness of this course?**	
Neutral	4 (10.0%)
Useful	16 (40.0%)
Very useful	20 (50.0%)
**Please rate your overall satisfaction towards this course**	
Neutral	4 (10.0%)
Satisfied	19 (47.5%)
Very satisfied	17 (42.5%)

**Fig. 1:**
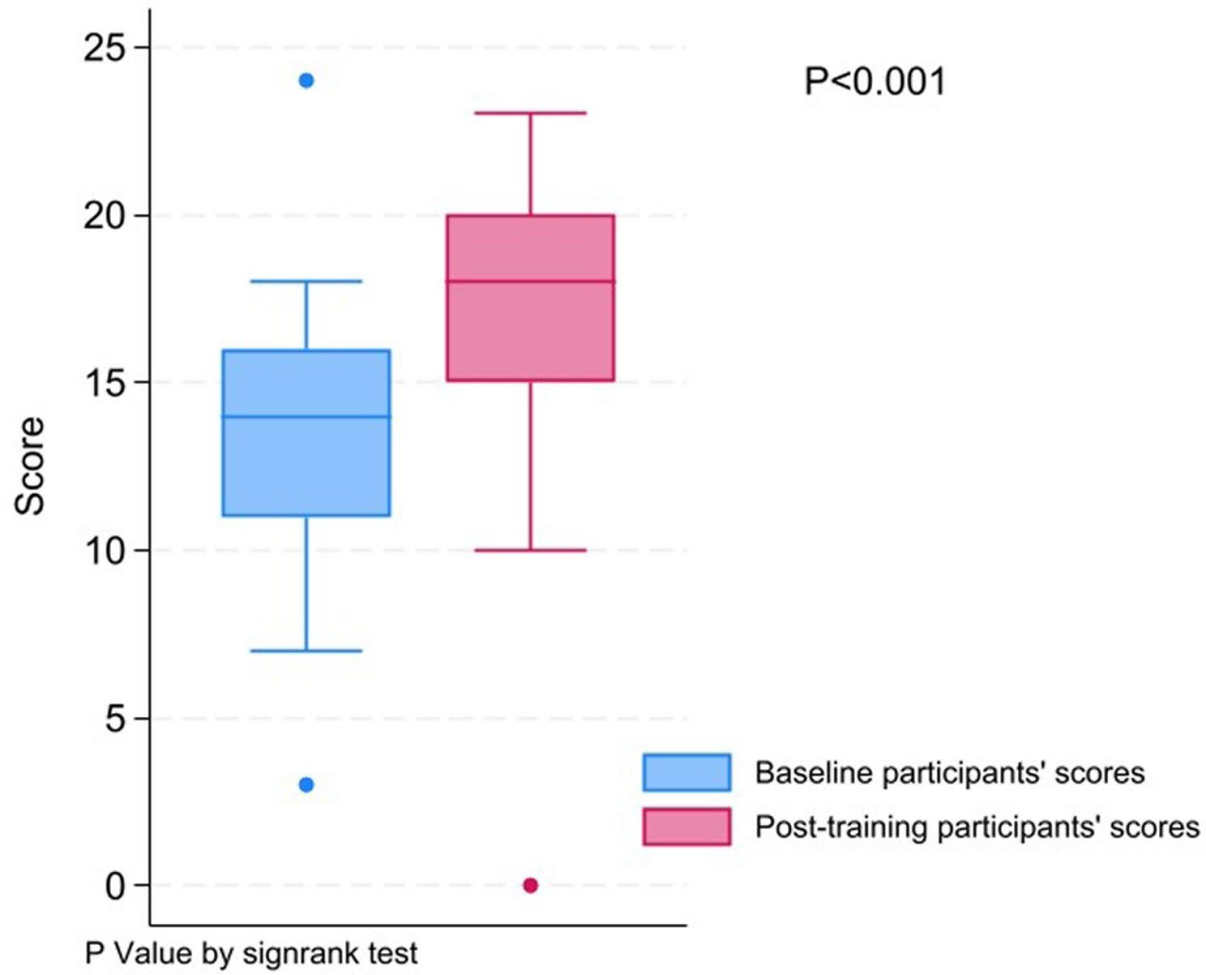
Participants’ overall knowledge scores (before and after the course)

Regarding participants’ overall knowledge scores, before the course, the median (IQR) knowledge score was 14(11:16). After completing the course, the median (IQR) knowledge score increased to 18 (15: 20. The difference in scores between the pre- and post-course assessments was statistically significant, with a p-value less than < 0.001 based on the sign rank test, as shown in Fig. 1.

**Fig. 2:**
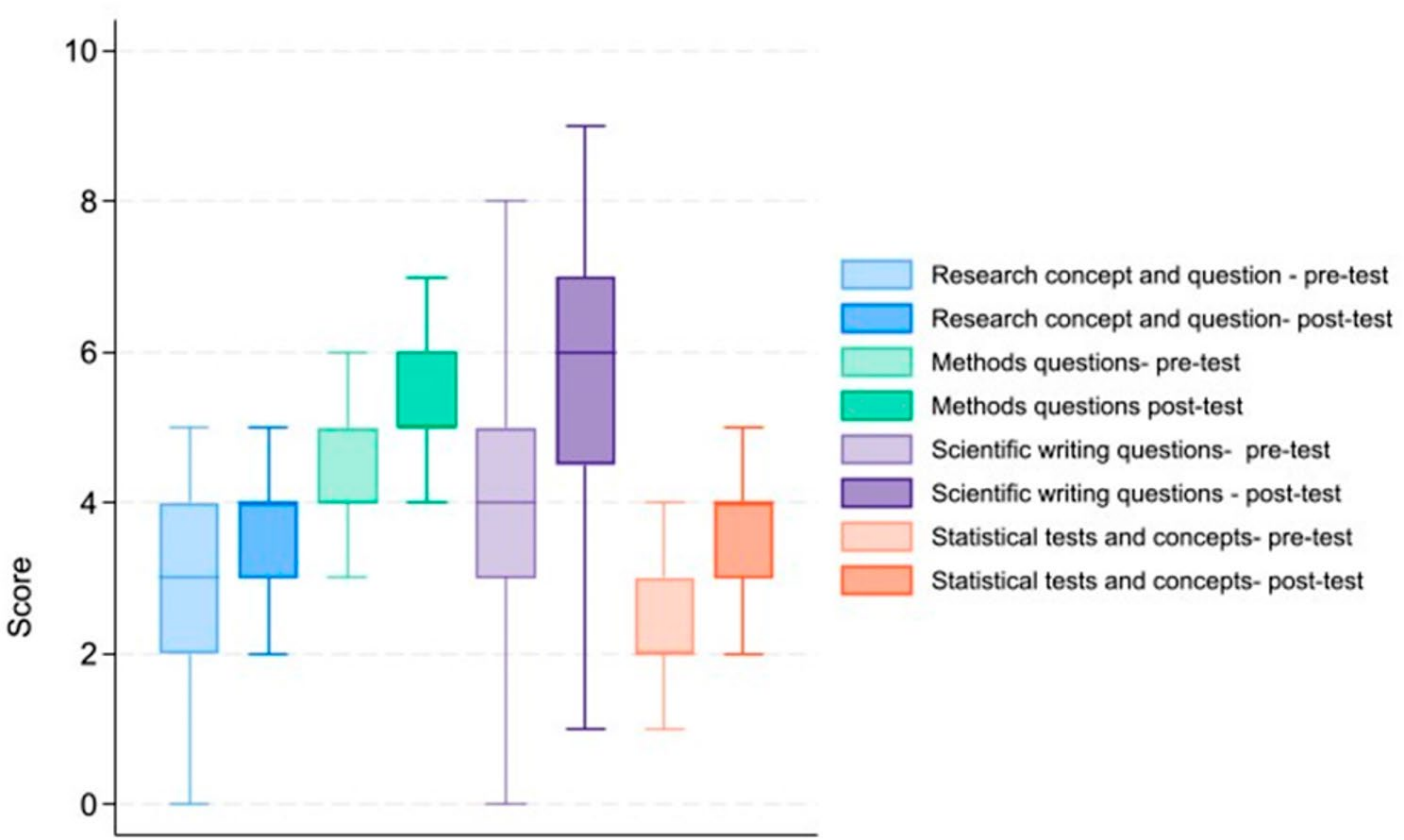
The four subgroups of knowledge scores

The four subgroups of knowledge scores related to research concept and question, methods and design, scientific writing, and biostatistics concepts were analyzed, and it was observed that three of the subgroups showed statistically significant improvement after the research training course. However, the subgroup specifically focused on research questions did not exhibit a significant improvement as shown in Fig. 2.

## Discussion

The findings of this study indicate a significant positive impact of a well-designed clinical research course on the knowledge and perception of family physicians, highlighting the practical benefits of structured research training in enhancing healthcare professionals’ competencies. This reflects the importance of continuous education and training in clinical research for healthcare professionals, which is essential for evidence-based practice. Unlike general research training programs, our course was tailored specifically for family physicians, addressing their unique needs in clinical research, a niche that has been relatively underexplored. These findings come in agreement with other studies [21–23] which also reported the positive impact of clinical research on knowledge gained.

Regarding characteristics of study participants in the research training course, most of participants were female, accounting for 93.9%, which aligns with the increasing representation of women in medical fields [24]. The split between MD and master’s degree candidates and the results that show statistically significant improvement in the knowledge score before and after the course reflect the importance of clinical research courses for healthcare professionals regardless of their scientific degree and training on clinical research to be continued throughout their career. The finding that 61.2% of participants were engaged in clinical research projects during the training course reflects the emphasis on integrating research into clinical practice so it is necessary to focus on training healthcare professionals on clinical research continuously throughout their careers.

The increase in overall knowledge scores, evidenced by a statistically significant improvement (*p* < 0.001) between pre- and post-course assessments, indicates the effectiveness of our structured approach to teaching fundamental research principles. While three subgroups—research concept and question, methods and design, and biostatistics concepts—demonstrated significant improvement, the lack of significant progress in the research questions subgroup warrants further exploration. Possible factors, such as the complexity of research questions or the need for additional targeted training, should be considered for future course refinement.

Comparing these findings to existing literature, our results align with studies emphasizing the efficacy of targeted clinical research training programs in improving knowledge and skills among healthcare professionals, such as the study of Sprague et al.,2012 that aimed to improve the knowledge of surgical residents and clinicians on the principles and practice of surgical research methodology in which overall participant knowledge about clinical research methods improved significantly from the pre- to the post-course test (mean improvement in score 13.5%, relative increase 35.3%, *p* < 0.001).^25^ However, our study adds to the literature by specifically addressing the training needs of family physicians and assessing not only knowledge gains but also practical skills and engagement in research activities.

The identification of “Scientific Writing” as the most useful module aligns with the recognized importance of effective communication in research [26]. 

The positive feedback on course content clarity and practical applicability is in line with studies emphasizing the importance of clear and practical training materials [25]. The satisfaction levels and willingness to recommend the course are consistent with research indicating that satisfaction is crucial for effective learning and knowledge transfer [27]. The participants’ satisfaction and willingness to recommend the course further validate its impact, reinforcing the role of well-structured training in fostering research engagement among family physicians.

The high percentage (90%) of participants finding the online format facilitated learning aligns with the growing recognition of the effectiveness of online education in healthcare [28]. This finding is particularly relevant in today’s digital learning landscape, suggesting that virtual training models can be leveraged to expand research education accessibility. The high percentage (92.5%) expressing confidence in applying gained knowledge and skills in future work aligns with the concept of competency-based education in medical training [29]. By equipping family physicians with research competencies, this course contributes to improving evidence-based practice, ultimately benefiting patient care and healthcare decision-making.

### Study limitation

There are some limitations to this study, which should be addressed in future research. First, we observed short-term improvements in knowledge that we cannot generalize to sustain longer-term knowledge about clinical research. Second, the relatively small sample size, also limits the generalizability of the findings. Third, our study did not include a control group, so it is hard to accurately evaluate the true impact of this research education intervention. Thus, more research on the long-term effectiveness of research training is needed, including a control group and a larger study sample.

## Conclusion

In conclusion, our study emphasizes the success of the designed course in enhancing the clinical research knowledge and perception of family physicians. The study provides valuable insights for educators and curriculum developers aiming to design similar courses. Further research can address potential areas for continued improvement in training programs.

## Data Availability

All data generated or analysed during this study are included in this published article.

## References

[1] Cole DC, Johnson N, Mejia R, McCullough H, Turcotte-Tremblay A-M, Barnoya J, et al. Mentoring health researchers globally: diverse experiences, programmes, challenges and responses. Glob Public Health. 2016;11(9):1093–108. 26234691 10.1080/17441692.2015.1057091 PMC5020346

[2] Khan H, Taqui AM, Khawaja MR, Fatmi Z. Problem-based versus conventional curricula: influence on knowledge and attitudes of medical students towards health research. PLoS ONE. 2007;2:7. 10.1371/journal.pone.0000632. 10.1371/journal.pone.0000632 PMC1913552 17637847

[3] Reinders JJ, Kropmans TJB, Cohen-Schotanus J. Extracurricular research experience of medical students and their scientific output after graduation. Med Educ. 2005;39:237. 10.1111/j.1365-2929.2004.02078.x. 15679693 10.1111/j.1365-2929.2004.02078.x

[4] Lucas M, Harrison RJ, Woods MC, McCarthy, Priti P, Parikh. Development and implementation of a sustainable research curriculum for general surgery residents: A foundation for developing a research culture. Am J Surg. 2020;220(1):105. 31590889 10.1016/j.amjsurg.2019.09.028

[5] Griffin MF, Hindocha S. Practices of medical students at British medical schools: experience, attitudes and barriers to publish. Med Teach. 2011;33:1–8. 21182368 10.3109/0142159X.2011.530320

[6] Ibrahim N, Fetyani D, Bashwari J. Assessment of the research-oriented knowledge, attitude and practice of medical students and interns of the King Abdulaziz university, Jeddah and the adoption of a research-intervention educational program. Rawal Med J. 2013;38:432–9.

[7] Devi V, Abraham RR, Adiga A, Ramnarayan K, Kamath A. Fostering research skills in undergraduate medical students through mentored student projects: example from an Indian medical school. Kathmandu Univ Med J. 2012;8:294–8. 10.3126/kumj.v8i3.6215. 10.3126/kumj.v8i3.6215 22610733

[8] Mullan JR, Weston KM, Rich WC, McLennan PL. Investigating the impact of a research-based integrated curriculum on self-perceived research experiences of medical students in community placements: a pre-and post-test analysis of three student cohorts. BMC Med Educ. 2014;14:161. 10.1186/1472-6920-14-161. 25096817 10.1186/1472-6920-14-161 PMC4134463

[9] Black ML, Curran MC, Golshan S, Daly R, Depp C, Kelly C, Jeste DV. Summer research training for medical students: impact on research self-efficacy. Clin Transl Sci. 2013;6:487–9. 10.1111/cts.12062. 24330695 10.1111/cts.12062 PMC3868994

[10] Jawaid M, Masood Z, Alam S, Jawaid S. An analysis of interactive hands-on workshops on medical writing. JPMA J Pakistan Med Assoc. 2011;66. 22368907

[11] Porta M. A dictionary of epidemiology. Oxford University Press; 2008.

[12] Emanuel EJ, Wendler D, Grady C. What Makes Clin Res Ethical? JAMA. 2000;283(20):2701–11. 10.1001/jama.283.20.2701 10819955

[13] Hulley SB, Cummings SR, Browner WS, Grady DG, Newman TB. Designing clinical research. Lippincott Williams & Wilkins; 2013.

[14] Dawson B, Trapp RG, Trapp RG. Basic & clinical biostatistics. Lange Medical Books/McGraw-Hill; 2004.

[15] Scales CD, Kupelian V. Improving data quality and management. JAMA. 2007;298(20):2433–4.

[16] DeRenzo EG, Moss J. Writing protocols for clinical research. JHU; 2002.

[17] European Medicines Agency. ICH Topic E 6 (R1) Guideline for Good Clinical Practice.2002.

[18] Vodopivec I, Vujaklija A, Hrabak M, Lukiæ IK, Marušiæ A, Marušiæ M. Knowledge about and attitudes towards science of first year medical students. Croat Med J. 2002;43:58–62. 11828562

[19] Khan H, Khawaja MR, Waheed A, Rauf MA, Fatmi Z. Knowledge and attitudes about health research amongst a group of Pakistani medical students. BMC Med Educ. 2006;6:54. 17081286 10.1186/1472-6920-6-54 PMC1635552

[20] Issrani D, Rakhi, Prabhu. Namdeo. Assessing knowledge, attitude and practices about clinical research among dental faculty and postgraduate students – an observational study. Int J Curr Adv Res. 2017;6:1811–15.

[21] Dako-Gyeke P, Asampong E, Afari E, Launois P, Ackumey M, Opoku-Mensah K, Dery S, Akweongo P, Nonvignon J, Aikins M. Capacity Building for implementation research: a methodology for advancing health research and practice. Health Res Policy Syst. 2020;18(1):53. 10.1186/s12961-020-00568-y. PMID: 32487176; PMCID: PMC7268492. 32487176 10.1186/s12961-020-00568-y PMC7268492

[22] Wang T, Barter S, Durieux M, Flickinger T, Twagirumugabe T, Banguti P. A qualitative evaluation of an operational research course for acute care trainees in Kigali, Rwanda. Pan Afr Med J. 2021;40:21. 10.11604/pamj.2021.40.21.29191. PMID: 34733389; PMCID: PMC8531958. 34733389 10.11604/pamj.2021.40.21.29191 PMC8531958

[23] Liira H, Koskela T, Thulesius H, Pitkälä K. Encouraging primary care research: evaluation of a one-year, doctoral clinical epidemiology research course. Scand J Prim Health Care. 2016;34(1):89–96. 10.3109/02813432.2015.1132893. Epub 2016 Feb 8. PMID: 26854523; PMCID: PMC4911026. 26854523 10.3109/02813432.2015.1132893 PMC4911026

[24] Penny M, Jeffries R, Grant J, Davies SC. Women and academic medicine: a review of the evidence on female representation. J R Soc Med. 2014;107(7):259–263. doi: 10.1177/0141076814528893. Epub 2014 Apr 16. PMID: 24739380; PMCID: PMC4093756. 10.1177/0141076814528893 PMC4093756 24739380

[25] Sprague S, Pozdniakova P, Kaempffer E, Saccone M, Schemitsch EH, Bhandari M. Principles and practice of clinical research course for surgeons: an evaluation of knowledge transfer and perceptions. Can J Surg. 2012;55(1):46–52. 10.1503/cjs.018610. PMID: 22269302; PMCID: PMC3270085. 22269302 10.1503/cjs.018610 PMC3270085

[26] Forero DA, Lopez-Leon S, Perry G. A brief guide to the science and Art of writing manuscripts in biomedicine. J Transl Med. 2020;18(1):425. 10.1186/s12967-020-02596-2. PMID: 33167977; PMCID: PMC7653709. 33167977 10.1186/s12967-020-02596-2 PMC7653709

[27] Vitéz N. László Duma, Measuring efficiency and effectiveness of knowledge transfer in elearning,Heliyon,Volume9,Issue7,2023,10.1016/j.heliyon.2023.e17502 10.1016/j.heliyon.2023.e17502 PMC10336452 37449147

[28] Reeves S, Fletcher S, McLoughlin C, et al. Interprofessional online learning for primary healthcare: findings from a scoping review. BMJ Open. 2017;7:e016872. 10.1136/bmjopen-2017-016872. 10.1136/bmjopen-2017-016872 PMC5623444 28780560

[29] Ten Cate O. Competency-Based postgraduate medical education: past, present and future. GMS J Med Educ. 2017;34(5):Doc69. 10.3205/zma001146. PMID: 29226237; PMCID: PMC5704607. 29226237 10.3205/zma001146 PMC5704607

